# Roll-out of an educational workshop to improve knowledge and self-confidence of healthcare professionals engaged in mainstreaming of breast cancer genetics

**DOI:** 10.1371/journal.pone.0307301

**Published:** 2024-07-19

**Authors:** Valerie Jenkins, Ruth Habibi, Virginia Hall, Pauline Leonard, Anneliese Lawn, Jay Naik, Rachel Papps-Williams, Lesley Fallowfield

**Affiliations:** 1 Sussex Health Outcomes Research & Education in Cancer (SHORE-C), Brighton & Sussex Medical School, University of Sussex, Falmer, United Kingdom; 2 Barking, Havering and Redbridge University Hospitals NHS Trust, London, United Kingdom; 3 Ashford & St Peter’s Hospitals NHS Foundation Trust, Surrey, United Kingdom; 4 Harrogate and District NHS Foundation Trust, Harrogate, United Kingdom; The University of Sydney, AUSTRALIA

## Abstract

**Background:**

There are calls worldwide for the mainstreaming of genetic testing in breast cancer (BC) clinics, but health care professionals (HCPs) are not always familiar with nor confident about genetic counselling. TRUSTING (**T**alking about **R**isk & **u**ncertaintie**s** of **T**esting **in G**enetics is an educational programme shown to significantly improve HCPs’ knowledge, communication, self-confidence, and self-awareness. We rolled out TRUSTING workshops across the UK and probed their influence on mainstreaming within BC clinics.

**Methods:**

1 surgeon, 3 oncologists, and 1 nurse specialist who had attended the original TRUSTING evaluation project were trained to facilitate the 8-hour programme in pairs. The participants (all health care professionals) attending their workshops completed 3 questionnaires: - 1) the Intolerance to Uncertainty Scale, 2) an 18-item multiple choice knowledge questionnaire about *BRCA* 1/2 gene testing, incidence and risk reducing interventions and 3) a 10-item questionnaire exploring self-confidence when advising patients and their families about these issues. Both knowledge and self-confidence were re-tested post workshop together with evaluation of the facilitators’ approach and overall satisfaction with the event. Follow-up questionnaires 3–12 months later examined impact of workshops on HCPs’ own practice and how mainstreaming was working in their clinics.

**Results:**

120 HCPs (61 surgeons; 41 nurses; 9 oncologists; 9 other) attended 12 workshops. Knowledge scores (mean change = 6.58; 95% CI 6.00 to 7.17; p<0.001), and self-confidence (mean change = 2.64; 95% CI 2.33 to 2.95; p<0.001) improved significantly post workshop. Ratings for the facilitators’ approach were uniformly high (mean range 9.6 to 9.9 /10). Most delegates found the workshops useful, enjoyable, and informative and 98% would definitively recommend them to colleagues. Follow-up data (n = 72/96) showed that 57% believed attendance had improved their own practice when discussing genetic testing with their patients. When asked about mainstreaming more generally, 78% reported it was working well, 18% had not yet started, and 3% thought it was problematic in their centre.

**Conclusions:**

Discussing the implications that having a pathogenic gene alteration has for patients’ treatment and risk-reducing interventions is complex when patients are already coming to terms with a breast cancer diagnosis. Training facilitators enhanced the wider roll-out of the TRUSTING educational programme and is an effective means of helping HCPs now involved in the mainstreaming of genetic testing.

## Introduction

Mainstreaming of genetic testing involves the ordering of genetic tests at point of care by relevant clinicians, rather than requiring pre-test referral to, and counselling by, a geneticist or genetic counsellor. The implementation of mainstreaming genetic testing in UK clinics started with ovarian cancer patients; this model appeared to be efficient, cost-effective, and relatively successful for patients and staff [1, 2].

Mainstreaming in UK breast cancer (BC) centres shortly after diagnosis is still relatively new and presents challenges for surgeons, oncologists, and specialist nurses. Explaining the risks, uncertainties and implications surrounding inherited BC genes is always difficult and members of a cancer team may not be especially experienced in genetics or in dealing with the sometimes challenging and complex conversations demanded [3]. All Health Care Professionals (HCPs) involved need to be able explain the rationale for testing and help support the patient if the genetic test is positive. They therefore need to be competent at addressing questions about the genetic test result simply enough for patients to consider their best management options and how these fit with their own personal preferences. This should focus predominately on treatment of the current BC whilst introducing issues such as appropriate prevention and risk reducing strategies. Navigating a discussion about the implications of potential hereditary risks with patients and other family members often takes place in an anxious, time pressured environment.

Resources are available that provide guidance and recommendations on genetic testing thresholds, surveillance and risk reducing strategies for people who already have or who are at risk of familial cancer including the National Institute for Health and Care Excellence (NICE) [4]. However, the guidance on page 112 states that “*testing and results could be given by any member of the multidisciplinary team* *if they have adequate training* *in the interpretation and communication of genetic test results*. *How the adequacy of knowledge level should be measured and assessed is unclear at present*, *in the absence of formal clinical genetics training*.*”* This is a potential gap and challenge for the smooth introduction of mainstreaming of BC genetics in clinics outside the main cancer centres. A nationwide survey (93% of UK oncologists) identified a need for additional genomics education, and although 81% reported a lack of education in genetic counselling, 49% had already consented patients for germline testing [5]. Merely having the appropriate genetic knowledge however is not sufficient, one needs to be able to communicate well, which can be difficult especially when patients are already coping with the stress and anxiety of a BC diagnosis.

Courses are available specifically aimed at increasing knowledge and communication about genetic testing; these may incorporate face to face small group work [6, 7] or are delivered online [8, 9]. Some have been shown to help but all have their strengths and weaknesses in terms of content or methods of evaluating efficacy.

The Massive Open Online Courses (MOOCs) in genomic variants interpretation for the National Health Service (NHS) workforce are described as educational resources comprising slides, videos from experts and patients’ stories [8]. Evaluation of the programme examined changes in knowledge and user reported self-confidence when managing variants of uncertain significance (VUS). Results showed improvements in both outcomes in a cohort of 92 HCPs (clinical scientists and non-genomics clinicians) [8]. However, numbers completing both MOOCs dropped from 92 (n = 66 genomics professionals; non-genomics clinicians n = 26), to 37 (32 and 5 respectively). Although there was increased learner confidence and a good ability to correctly classify genomic variants, participants’ prior knowledge was unknown. The courses were described as engaging and relevant to their professional development but more of the non-genomic clinical staff rated the content as too complex, and others wanted more interaction and feedback.

Another online training programme with 32 HCPs reported positive changes in genetic literacy and competence in communication [9]. The content was based on previously developed face to face training and included two modules: 1) introduction to mainstream genetic testing and 2) the mainstream genetic testing process. The modules included eight embedded videos featuring a genetic counsellor, oncology nurse and medical oncologist. The website also contained links to resources such as the Australian national guidelines on *BRCA 1 / 2* testing and patient education leaflets. Participants felt they had gained new skills and increased confidence when communicating with patients. However, as with the MOOCs there was no baseline measure of knowledge or confidence prior to starting the training. Additionally, around a quarter either ‘did not believe’ or were ‘unsure’ if the videos were useful. This led the authors to conclude that *“an important aim of future research should be to develop communication skills training programs specific to cancer genetics incorporating experiential learning activities”* [9].

The only study to date that evaluated communication objectively pre and post workshop was the TRUSTING (Talking about Risk and UncertaintieS of Testing IN Genetics) educational programme [6]. It incorporates both a traditional and learner centred approach. The traditional aspect is the didactic presentation containing research on the communication of risk, health literacy and numeracy, together with interactive exercises. The learner centred approach based on Lipkin’s educational model [10], is where adults are encouraged to actively engage and take responsibility for their own learning. The programme is facilitated in small groups (8–12 participants per workshop) and comprises six scenarios. These follow the proband (first person in the family to be identified as possibly having a genetic disorder) who is diagnosed with Triple Negative Breast Cancer (TNBC) and later learns she is *BRCA 2* positive, her sister who tests positive, and an anxious cousin who tests negative. Each filmed scenario features a non-scripted consultation with a real-life HCP and the “actor” patient, or member of her family. There is also an interview with a geneticist who provides much of the knowledge-based information required when providing relevant information. The 8-hour workshops cover 1) research-based evidence about ways to present complex risk information, 2) ethics, 3) communication about family history & what genetic testing means for family members, 4) discussing *BRCA* test results and implications for gene mutation carriers and those who test negative, and 5) impact of health literacy and numeracy on decision-making.

Following the published positive evaluation [6] there was a demand from many other HCPs to attend TRUSTING workshops across the UK. To enable a swift cascade of the programme we needed to train more facilitators to conduct further workshops. The aim of the study was to assess 1) wider utility of the materials in terms of improving confidence and knowledge when facilitated by surgical and oncology professionals and 2) if learning enhanced the self-reported clinical practice of participants engaged in mainstreaming. The results of these workshops are presented in this paper.

## Materials and methods

**Fig 1:**
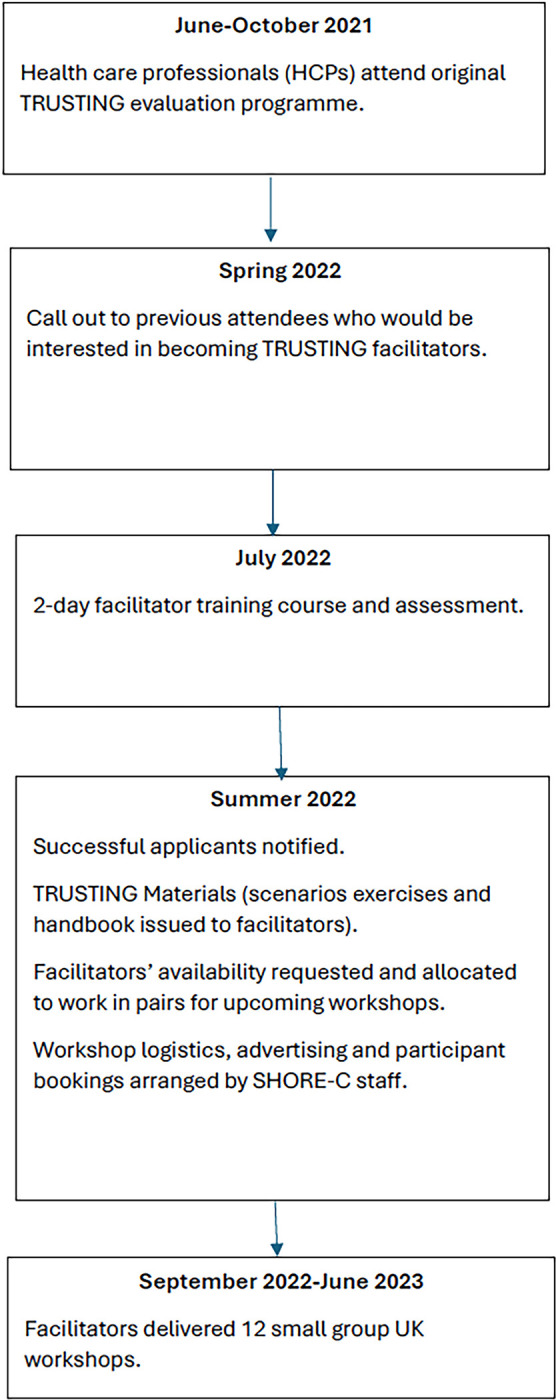
Schema of steps involved in facilitator selection and TRUSTING workshops.

There are two parts to the methods: 1) selection and training of the facilitators and 2) facilitation of the TRUSTING workshops. For ease of reference see Fig 1.

### Selection and training of the facilitators

Surgeons, nurses, and oncologists who participated in the original TRUSTING evaluation course [6] indicated their interest in learning how to facilitate the educational modules. Seven applied and attended a 2-day facilitator training course, which included how to utilise a learner centred approach (based on [10]) and ensure group confidentiality, and demonstrations about handling group conflict. Prior to attendance the trainees were allocated a specific scenario to familiarise themselves with it in order to practice facilitation on the course. Their performance was assessed by 2 of the authors (LJF and VJ) using a 16-item Core Competency checklist comprising items such as dealing with practical issues (e.g. seating arrangements, time keeping, handling of equipment), through to awareness of group dynamics, and encouraging contributions from all participants. Five trainees scored ‘very good’ to ‘excellent’ in at least 12/16 core competencies and were therefore invited to be TRUSTING facilitators (3 oncologists, a surgeon and family history nurse). Three already had previous experience of facilitating small groups with different educational materials produced by the authors LJF and VJ and two were new to this type of format. It was decided therefore that a more experienced and a new trainee would facilitate twelve TRUSTING workshops in pairs. Each facilitator was issued with resource materials:- 1) USB that contained the 6 scenarios plus an interview with geneticist Professor Gareth Evans, 2) a comprehensive timecoded handbook with transcripts of all the interviews and suggestions for stopping points and prompts for group discussions, 3) a PowerPoint presentation about risk and uncertainty and 4) copies of all the interactive exercises used during a workshop including basic numeracy, Intolerance of Uncertainty, and explaining frequencies.

### Organisation of TRUSTING workshops and recruitment of delegates

To minimise the administrative burden associated with conducting regional residential (optional) workshops, staff at the research unit (Sussex Health Outcomes Research & Education in Cancer–SHORE-C) organised and advertised the course dates, venues, CPD accreditation, and interacted with potential delegates. They also obtained ethical approval and ensured delegates had completed assessments prior to the workshop. The facilitators were sent questionnaires and exercises to administer during the workshops which were returned securely to SHORE-C for analysis.

### Inclusion criteria

Health care professionals actively engaged in discussing *BRCA1* and *BRCA2* genetic testing and/or the results in a breast cancer or genetics service setting were eligible to attend a TRUSTING workshop.

### Workshop assessments

The measures were used previously in the original TRUSTING evaluation study [6]: -

1. the 12-item Intolerance of Uncertainty Scale (IOU) (12–60 score) [11] that provides participants with insights about their own tolerance to uncertainty. Individuals with a high score are often risk averse and may convey their own intolerance to uncertainty through nuanced communication with their patients.
2. the 4-item numeracy scale based on Schwartz [12] examines participants’ ability to convert probabilities into proportions, proportions into percentages and vice versa; important skills to enable better patient understanding of risk.
3. a 10-item self-confidence questionnaire about discussing risk and uncertainties in breast cancer genetics and
4. an 18-item Multiple Choice Questionnaire (MCQ) to assess knowledge about genetic testing in people with and without BC.

The IoU and self-confidence questionnaire were completed on-line prior to attendance and the data used in a didactic presentation relevant for each group. The knowledge MCQ was administered at the workshop before viewing any of the scenarios. Numeracy exercises were completed at appropriate points during discussions about the importance of being flexible when helping patients understand and retain important numbers describing risk. The knowledge MCQ, and self-confidence assessments were repeated at the workshop conclusion. Delegates rated the quality and abilities of the facilitators, the educational materials, and whether or not they would recommend workshops to colleagues. Delegates were sent another questionnaire (1–3 months post workshop) exploring the impact attendance had had on their own clinical practice, and how mainstreaming of genetic testing was working at their cancer centre.

The Royal College of Physicians accredited the workshops with 9 Continuing Professional Development (CPD) points. The TRUSTING materials were developed via a research grant from the Breast Cancer Research Foundation. Facilitator training and workshops via an educational grant from AstraZeneca. Written informed consent was obtained for all participants and Brighton & Sussex Medical School Research Governance and Ethics Committee approved the study (ref: ERA/RMLS21/8/2).

## Results

Between September 2022 and June 2023, 12 facilitator-led face-to-face workshops were attended by 120 HCPs (see Table 1), of whom 36 held senior posts.

**Table 1:** Workshop participant characteristics.

Characteristics	N (120)
**Gender**	
Male; Female	26; 94
**Job role**	
Consultant Surgeon	28
Non consultant surgeon	33
Nurse	41
Consultant Oncologist	6
Non consultant oncologist	3
Consultant Other	2
Other	7
**Area of UK**	
England	104
Scotland	6
Wales	8
Northern Ireland	2

### Self-confidence

#### Primary problems areas identified by participants’ pre-workshop

Attendees felt less confident when dealing with those individuals who had an anxious predisposition (44/120; 36.67%). Other issues found challenging included:—discussing indeterminate findings and advising individuals who are pathogenic gene variant carriers about continuation of HRT.

**Fig 2:**
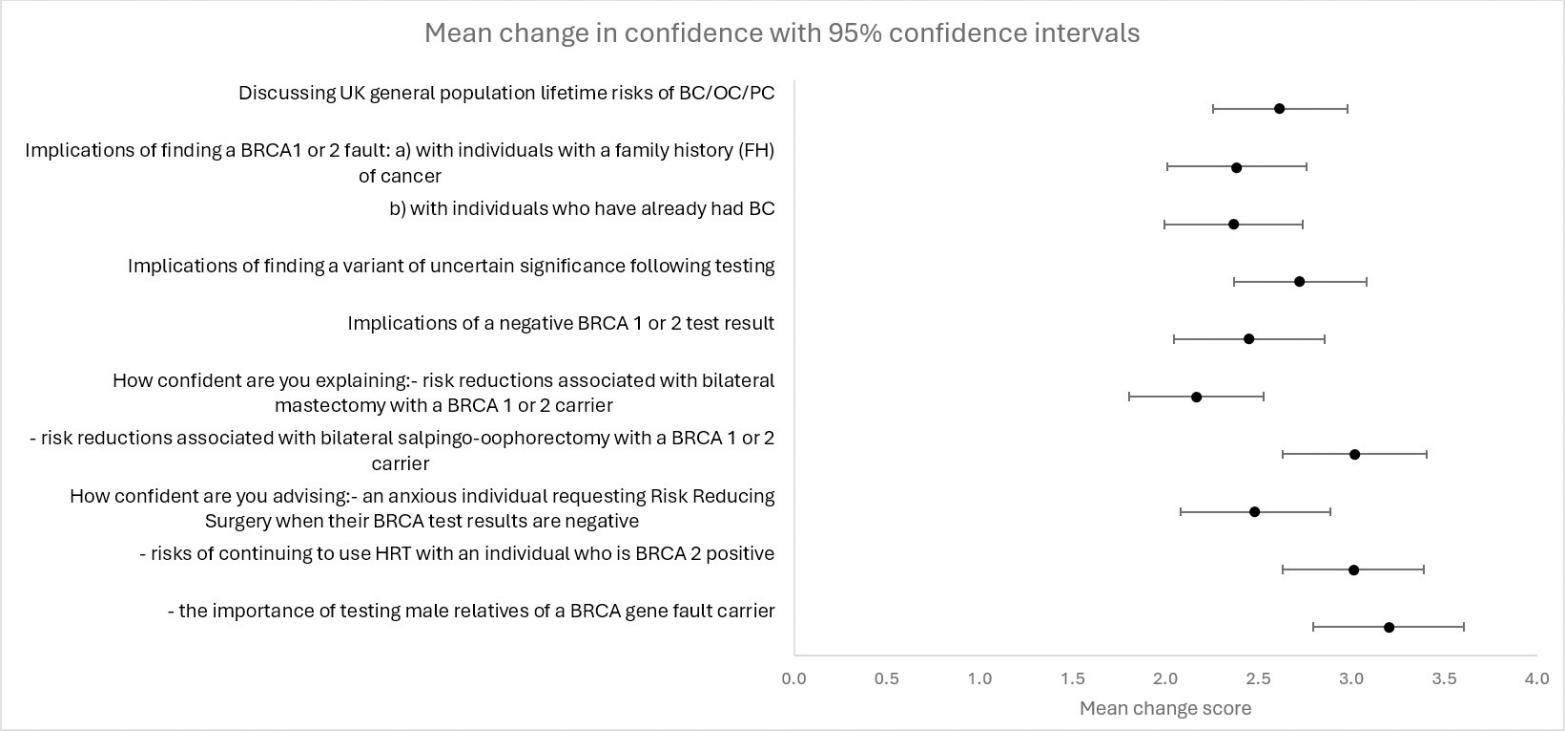
Forest plot showing changes in participants’ self confidence.

Fig 2 (Forest plot) shows that participants’ self-confidence when communicating about various issues concerned with genetic testing and treatment implications all improved significantly post-workshop (mean change = 2.64; 95% CI 2.33 to 2.95; p<0.001). Improvements were seen irrespective of attendees’ specialty.

#### Intolerance of Uncertainty Scale (IUS)

IUS scores by specialty are shown in Table 2. As a group, participants had mean (sd) scores similar to norms (29.53 (10.96) [11]. This suggests a reasonable tolerance of uncertainty (surgeons 30.54 (8.36) specialist nurses 26.32(6.63), oncologists 31.44 (5.15) and other 30.44 (12.99). There were no significant differences between the specialties but 12 had total scores consistent with generalised anxiety disorder (score >39).

**Table 2:** Intolerance of uncertainty–Mean (sd) scores for workshop participants.

	Surgeons (n = 61)	Nurses (n = 41)	Oncologists (n = 9)	Others (n = 9)
Prospective Anxiety (0–35)	20.03 (5.29)	16.66 (4.06)	19.44 (3.81)	18.78 (6.74)
Inhibitory Anxiety (0–25)	10.51 (4.02)	9.66 (3.10)	12.00 (2.12)	11.67 (6.48)
Total (0–60)	30.54 (8.36)	26.32 (6.63)	31.44 (5.15)	30.44 (12.99)

#### Basic numeracy

Table 3 shows the percentage of participants who gave correct responses to the 4 basic numeracy items. The question that caused the most challenge was item 3, which required calculating a number from a fraction, with lowest scores for all specialties.

**Table 3:** Basic numeracy—Percentage of workshop participants answering correctly.

	Surgeons (n = 61)	Nurses (n = 41)	Oncologists (n = 9)	Others (n = 9)
A person taking Drug A has a 1% chance of an allergic reaction. If 1000 people take the drug how many will have a reaction?	95.1%	73.2%	100.0%	77.8%
A person taking Drug B has a 1 in a 1000 chance of an allergic reaction. What % of people taking the drug will have a reaction?	85.2%	41.5%	66.7%	55.6%
The chance of getting a serious viral infection is 0.0005. How many of 10,000 exposed people might get the infection?	68.9%	34.2%	66.7%	55.6%
Imagine I flip a fair coin 1000 times. How many times will the coin land heads up?	88.5%	65.9%	100%	100%

#### Knowledge scores

Table 4 shows participants’ knowledge scores. Irrespective of specialty total scores improved for all, the mean change post-workshop was 6.58; 95% CI 6.00 to 7.17; p<0.001.

**Table 4:** Knowledge results.

	Surgeons (n = 61)		Nurses (n = 41)		Oncologists (n = 9)		Others (n = 9)	
Question	Before	After	Before	After	Before	After	Before	After
What proportion of the UK general population carry a BRCA 1 or BRCA 2 fault?	14.8% (9/61)	95.1% (58/61)	22% (9/41)	82.9% (34/41)	44.4% (4/9)	88.9% (8/9)	55.6% (5/9)	100% (9/9)
What proportion of all UK breast cancer cases is caused by an inherited BRCA 1 or BRCA 2 fault?	26.2% (16/61)	91.8% (56/61)	19.5% (8/41)	82.9% (34/41)	22.2% (2/9)	88.9% (8/9)	11.1% (1/9)	77.8% (7/9)
What proportion of the Ashkenazi Jewish population have a fault in BRCA 1 or BRCA 2 genes?	36.1% (22/61)	90.2% (55/61)	39% (16/41)	80.5% (33/41)	66.7% (6/9)	100% (9/9)	44.4% (4/9)	77.8% (7/9)
What proportion of all triple negative BC are due to a BRCA1 or BRCA 2 fault?	37.7% (23/61)	21.3% (13/61)	46.3% (19/41)	22% (9/41)	44.4% (4/9)	0% (0/9)	22.2% (2/9)	22.2% (2/9)
Which gene fault has a higher lifetime risk of ovarian cancer?	45.9% (28/61)	55.7% (34/61)	43.9% (18/41)	56.1% (23/41)	44.4% (4/9)	33.3% (3/9)	55.6% (5/9)	77.8% (7/9)
Ovarian cancer in women < 60yrs with no family /personal history of breast cancer is more likely to be BRCA 2 than BRCA 1	39.3% (24/61)	77.0% (47/61)	26.8% (11/41)	63.4% (26/41)	66.7% (6/9)	66.7% (6/9)	11.1% (1/9)	88.9% (8/9)
At what age do UK guidelines suggest MRI breast screening be considered for high-risk women?	93.4% (57/61)	93.4% (57/61)	56.1% (23/41)	87.8% (36/41)	55.6% (5/9)	77.8% (7/9)	55.6% (5/9)	88.9% (8/9)
According to UK guidelines what does the 10yr risk of developing BC in young women have to be before annual MRI screening can start?	29.5% (18/61)	55.7% (34/61)	12.2% (5/41)	34.1% (14/41)	11.1% (1/9)	44.4% (4/9)	55.6% (5/9)	66.7% (6/9)
What are the lifetime risks of developing BC for a post-menopausal woman with a BRCA 2 gene fault and a family history of BC in her?	36.1% (22/61)	78.7% (48/61)	43.9% (18/41)	85.4% (35/41)	33.3% (3/9)	66.7% (6/9)	33.3% (3/9)	88.9% (8/9)
What does SNP stand for?	49.2% (30/61)	86.9% (53/61)	19.5% (8/41)	68.3% (28/41)	88.9% (8/9)	100% (9/9)	55.6% (5/9)	77.8% (7/9)
What is the UK lifetime risk of prostate cancer for men with a BRCA 2 gene fault?	36.1% (22/61)	91.8% (56/61)	29.3% (12/41)	87.8% (36/41)	33.3% (3/9)	88.9% (8/9)	33.3% (3/9)	100% (9/9)
How much does 10 years of combined HRT, increase the risk of BC in women who are BRCA 2 positive?	60.7% (37/61)	100% (61/61)	65.9% (27/41)	97.6% (40/41)	55.6% (5/9)	100% (9/9)	55.6% (5/9)	100% (9/9)
Which one of these in general is most likely to increase BC risk in women?	18% (11/61)	90.2% (55/61)	29.3% (12/41)	82.9% (34/41)	11.1% (1/9)	88.9% (8/9)	11.1% (1/9)	77.8% (7/9)
Would you advise her (Anna) to have risk reducing bilateral mastectomy?	68.9% (42/61)	85.2% (52/61)	68.3% (28/41)	78% (32/41)	55.6% (5/9)	100% (9/9)	55.6% (5/9)	100% (9/9)
Which surgical risk-reducing management strategy would you suggest Josephine prioritises?	27.9% (17/61)	65.6% (40/61)	22% (9/41)	87.8% (36/41)	22.2% (2/9)	77.8% (7/9)	44.4% (4/9)	88.9% (8/9)
Josephine has been taking combined HRT for 3 years, would your initial advice be to: a) stop taking it now; b) continue with oestrogen only HRT c) not worry at the moment	9.8% (6/61)	32.8% (20/61)	14.6% (6/41)	48.8% (20/41)	0% (0/9)	11.1% (1/9)	11.1% (1/9)	44.4% (4/9)
Despite the negative BRCA result, is Emma’s lifetime risk of developing BC: a) same as the general population; b) moderately higher than the general population; c) much higher than the general population	55.7% (34/61)	91.8% (56/61)	48.8% (20/41)	78% (32/41)	33.3% (3/9)	88.9% (8/9)	55.6% (5/9)	88.9% (8/9)
At what age can she (Emma) start having mammographic screening?	70.5% (43/61)	98.4% (60/61)	51.2% (21/41)	100% (41/41)	11.1% (1/9)	100% (9/9)	55.6% (5/9)	100% (9/9)

NB. These last 5 questions relate to scenarios shown in the workshops

### Evaluation of workshops

Participants rated workshops highly—mean score of 9.6/10 for usefulness, 9.6 informative and 9.7 enjoyment, and all would recommend them to their colleagues. Every facilitator received high ratings for their general approach (9.8/10), handling group discussions and dynamics (9.9), familiarity with equipment (9.7) and scenarios (9.9), providing opportunities for all to contribute (9.9), and time keeping (9.9). They also provided written feedback, and made suggestions for future modules, including adding other scenarios about genetic testing in prostate and pancreatic cancer.

“*This is probably the most useful and applicable course I have ever attended. The benefits for me have gone beyond the subject matter of genetic risk to include more general aspects of consultations skills. I found the format of "sitting in" on consultations extremely valuable and think it should be used more often in training.”* Surgeon

### Follow-up mainstreaming questionnaire

72/96 (75%) participants completed the 6-item follow-up questionnaire. Over half (57%, 41) said the workshop had a significant impact on their own practice (36%, 26 modest impact; 7%, 5 negligible).

“*Seeing more BRCA mutation pts and counselling more confidently”* Oncologist

“*I had a very good conversation with one particular patient about risk to her family and it was all because of this workshop*. *It has also helped me stratify risk in other patients and included colorectal and prostate cancer in this”*. Surgeon

“*The workshop really improved my confidence that I am practicing well with good knowledge of the topic*. *Thank you again*!*”* Genetic Counsellor

The majority reported that mainstreaming of genetic testing was working well (56, 78%). However 2 (3%) viewed it as problematic and 13 (18%) said that it had not started at their centre, often due to low staffing levels.

“*There are a number of blocks currently. Wider MDM understanding and buy in, funding provision and (by extension) available workforce are all significant barriers currently but hopefully this will start to change soon.”* Nurse

Participants also provided thoughts and suggestions for future materials and workshops. These included scenarios on gene alterations, treatment and risk reducing implications for prostate cancer, Lynch syndrome, and pre-implementation genetic testing. They valued the format of the workshop using filmed scenarios and group discussion (82% 59/72) and some believed a second didactic presentation on current guidance and facts would be useful together with take-home summary sheets with references.

## Discussion

The results show that the TRUSTING educational programme materials delivered by health professionals, two of whom were new to group facilitation, produced significant improvements in knowledge and self-confidence for HCPs working in breast cancer. The findings were equally as positive as the original workshops conducted by the developers (LJF, VJ) [6] which reflects the wider utility of the materials, in addition to the facilitators’ competency.

Participants’ initial pre-workshop self-confidence ratings were lowest for 1) discussing variants of uncertain significance (VUS) following testing 2) explaining risk reductions associated with bilateral salpingo-ophorectomy, and 3) advising a BRCA 2 positive individual about her risk of continuing to use HRT. These changed significantly post workshop, items 2 and 3 were covered in a scenario and the amount of change was greater than for discussing VUS, which did not feature in any scenario in detail.

The follow-up questionnaire data showed that a few participants were experiencing issues starting mainstreaming at their centre. Their free text comments described how knowledge gained at the workshops had directly influenced the way they discussed the implications associated with BRCA gene testing and/or presented the results from gene testing. Results suggest that workshop attendance appeared to influence clinical practice, although more research is needed to explore this in detail and assessing objective change in clinical behaviours can be time consuming and costly. Nevertheless, there is evidence from other work using a similar educational format, which shows that complex communication skills can be taught and that if taught well do transfer into the clinic setting [13]. The three essential components required for this to occur are: - 1) elements that expand or solidify participants’ knowledge base, 2) communication skills development and 3) personal awareness [13].

However, direct evidence for the workshops effecting change concerning mainstreaming of genetics in their clinics was not robustly examined and needs further investigation. For some mainstreaming was already in place prior to workshop attendance. There were also several conflicting opinions from HCPs working in the same clinics as to whether or not introducing mainstreaming was problematic.

Successful mainstreaming of genetics/genomics may depend upon the degree of inter-professional communication that exists at individual sites. Although the TRUSTING programme was effective, more innovative methods might be required to efficiently target larger groups of HCPs. Online and E- learning approaches are successful ways to improve HCPs’ genetic knowledge at a self-chosen time [8, 9]. Others have incorporated interpersonal communication skills in their programmes, but this was in small group work [7]. Using simulated (actor) patients for role play is expensive and time consuming but often highly effective [13, 14]. A potential solution that is receiving much attention in medical education is the use of Virtual Reality (VR) to navigate challenging conversations [15], yet mixed attitudes were reported from nurses who engaged in a Virtual Counseling study [16]. Whilst the nurses recognised the potential benefits of VR, they found the virtual patients inauthentic. Currently underway in our SHORE-C unit is a pilot study with final year medical students to assess the feasibility and acceptability of using VR training module to navigate angry conversations [17] BodySwaps. The students will either receive the intervention via a desktop application or via a VR headset. If this method proves successful, there could be scope to move into other challenging areas such as discussing *BRCA* 1/ *BRCA* 2 testing with patients and their families.

## Conclusion

The results from this study suggest that the TRUSTING educational materials, when delivered by skilled facilitators in small group workshops, significantly improves the knowledge and self-confidence of HCPs engaged in mainstreaming of breast cancer genetics. Previous work has already shown objective improvements in communication with patients and individual comments made by participants in the study reported here attest to the benefits attendance at a TRUSTING workshop had on their interactions in their own clinical practices. Mainstreaming is likely to feature in all cancer centres so a requirement for extra training for non-geneticists may be mandatory.
